# NAPRT, but Not NAMPT, Provides Additional Support for NAD Synthesis in Esophageal Precancerous Lesions

**DOI:** 10.3390/nu14224916

**Published:** 2022-11-20

**Authors:** Niannian Wang, Da Pan, Xuemei Wang, Ming Su, Xin Wang, Qingyang Yan, Guiju Sun, Shaokang Wang

**Affiliations:** 1 Department of Nutrition and Food Hygiene, School of Public Health, Southeast University, Nanjing 210009, China; 2 Huai’an District Center for Disease Control and Prevention, Huai’an 223001, China; 3 Department of Public Health, School of Medicine, Xizang Minzu University, Xianyang 712000, China

**Keywords:** NAD, NAPRT, NAMPT, PARP-1, esophageal precancerous lesions

## Abstract

It is hypothesized that esophageal precancerous lesions (EPLs) have a surge requirement for coenzyme I (NAD). The purpose of this study is to clarify the key control points of NAD synthesis in developing EPL by detecting related markers and the gene polymorphism of NAD synthesis and metabolism. This case–control study was conducted in Huai’an, China. In total, 100 healthy controls and 100 EPL cases matched by villages, gender, and age (±2 years) were included. The levels of plasma niacin and nicotinamide, and the protein concentration of NAMPT, NAPRT, and PARP-1 were quantitatively analyzed. PARP-1 gene polymorphism was detected to determine if the cases differed genetically in NAD synthesis. The levels of plasma niacin and nicotinamide and the concentrations of NAMPT were not related to the risk of EPL, but the over-expressions of NAPRT (*p* = 0.014, 0.001, and 0.016, respectively) and PARP-1 (*p* for trend = 0.021) were associated with the increased EPL risk. The frequency distribution of APRP-1 genotypes was found to not differ between the two groups, while the EPL group showed an increased frequency of the variant C allele. NAPRT, but not NAMPT, was found to be responsible for the stress of excess NAD synthesis in EPL. Focusing on the development of NAPRT inhibitors may be beneficial to prevent and control ESCC.

## 1. Introduction

Esophageal squamous carcinoma (ESCC) is the most common subtype of esophageal cancer, cases of which are mainly found in East Asia, especially in China [1]. ESCC develops from esophageal precancerous lesions (EPLs), which are characterized by the dysplasia of esophageal mucosal cells. Esophageal cancer is generally not detected until the late stage because its early symptoms are not obvious, which leads to a poor prognosis and high mortality, even after treatment [2]. Fortunately, the EPL can be delayed or even reversed with advisable interventions [3]. Therefore, it is important to investigate the changes in key compounds in specific biochemical reactions occurring during EPL in vivo for further effective prevention of ESCC. The role of coenzyme I (NAD, also known as nicotinamide adenine dinucleotide) is of note.

As a coenzyme in metabolic pathways, NAD acts as an electron acceptor/donor during redox reactions occurring within cells and provides support for energy production [4]. A total of three pathways are present for the synthesis of NAD: the de novo pathway, the Preiss–Handler (PH) pathway, and the salvage pathway. The de novo pathway enzymes are restricted to some extent because they are mainly expressed in the liver and kidney [5]. Therefore, the latter two pathways are mainly responsible for NAD generation. The PH pathway begins with niacin, which generates NAD through the key role of the rate-limiting enzyme nicotinic acid phosphoribosyltransferase (NAPRT) [5,6,7]. Whereas, in the salvage pathway, it is the nicotinamide phosphoribose transferase (NAMPT) that is rate-limiting in nicotinamide consumption [8].

Due to the space-free growth pattern of cancer cells and the corresponding Warburg effect, there is a surging demand for higher production of NAD, as well as for substrates and rate-limiting enzymes in the main synthesis pathway [9,10]. Experiments in vitro found that NAPRT is overexpressed in esophageal cancer [11,12]. In addition, Takahashi et al. detected a significant upregulation in NAMPT mRNA expression in the serum of esophageal cancer patients during the perioperative period [13]. Currently, NAMPT has received more attention in cancer treatment [14,15]. NAMPT inhibitors have demonstrated antitumor activity in certain cancer cells, but it is not known whether they can achieve the same effect in esophageal cancer [15,16,17]. 

In addition, NAD also plays a cellular regulatory function as a substrate for other enzymatic reactions, including that catalyzed by poly ADP-ribose polymerase (PARP), which is essential for the repair of DNA in cancer cells [18,19]. PARPs use NAD as the source of ADP-ribose groups to synthesize protein complexes in combination with broken DNA strands. Activated PARPs, mainly PARP-1, bind to specific proteins through a series of long-branched ADP-ribose polymers (PARs) to repair DNA and control downstream reactions to DNA damage [20,21]. Cancer cell DNA damage is frequent, and to maintain cell survival, higher activity of PARPs is usually relied on [19,22]. The same applies to EPL, which was found to express abundant DNA damage variants in tissues [23].

However, the activity of PARPs does not only depend on the stock of NAD in vivo. Zhang et al. found that there is an exchange of valine (Val) to alanine (Ala) in the catalytic domain of PARP-1 in exon 17. The protein concentration of PARP-1 may be altered as a result, resulting in an increase in susceptibility to cancer. [24]. Three meta-analyses have consistently shown that the PARP-1 V762A polymorphism is associated with cancer risk in Asian populations [21,25,26].

The next-generation sequence analysis of EPL and ESCC tissues revealed a high similarity in gene mutations and copy-number alterations, proving that the initial ESCC clone forms early in the EPL stage [23]. Therefore, it is likely that the supply of NAD, as well as the PARP-1 genetic phenotype, is equally important for EPL. Understanding the characteristics of NAD synthesis and PARP-1 gene polymorphism in EPL can provide ideas for the prevention and control of ESCC. To this end, we conducted a matching 1:1 case–control study in Huai’an district, a region with a high prevalence of ESCC in China, and tested the blood samples of 100 pairs of study subjects.

## 2. Materials and Methods

### 2.1. Study Population and Sample Collection

The study population came from The Early Diagnosis and Early Treatment Project of Esophageal Cancer. The recruitment of subjects has been described in detail in other studies [27,28].

In total, 100 cases with mild or moderate EPL were randomly selected, and 100 healthy subjects without esophageal dysplasia from EDETPEC were matched into the control group. The two groups of subjects were matched according to region, sex, and age (±2 years). Epidemiological data and dietary circumstances were obtained by surveys and published [27]. The collection and preservation of the blood samples from 200 subjects were also described in detail previously [28].

### 2.2. Laboratory Measurements

The enzyme-linked immunosorbent assay (ELISA) (Nanjing Jin Yibai Biological Technology Co., Ltd., Nanjing, China) was used to determine the levels of niacin and nicotinamide and the activities of related enzymes in plasma samples. Testing was performed in strict accordance with the recommended procedures from the manufacturer. The OD values were measured at the wavelength of 450 nm using a microplate reader (Tecan Trading Co., Ltd., Shanghai, China).

**Figure 1:**
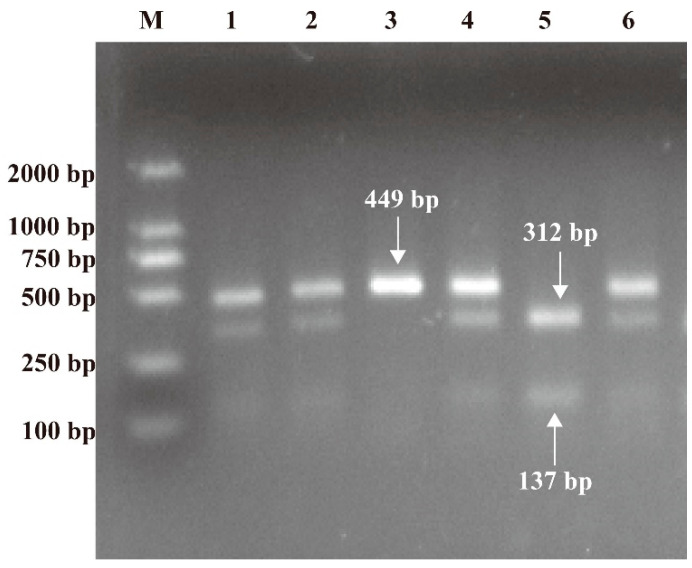
RFLP photograph of 2% agarose gel electrophoresis representing NAPRT V762A polymorphism. Lane 3 was characterized by a single 449 bp representing wild genotype TT; lanes 1, 2, 4, and 6 were characterized by 449, 312, and 137 bp representing heterozygote TC; lane 5 was characterized by 312 and 137 bp representing variant genotype CC; lane M represents the DNA marker.

The Wizard^®^ Genomic DNA Purification Kit (Promega, Madison, WI, USA) was used to extract genomic DNA on serum samples. Polymerase chain reaction–restriction fragment length polymorphism (PCR-RFLP) was used to evaluate PARP-1 V762A gene polymorphism with sense primer 5′-TGGCTCAGGACCCATTTGTC-3′ and antisense primer 5′-GAAGGCCTGACCCTGTTACC-3′. The one showing a band of 449 bp is the wild genotype TT. The heterozygous TC shows three bands of 449, 312, and 137 bp, while the one with two bands is the variant genotype CC with 312 and 137 bp, respectively. PCR amplification was performed with a PCR premix kit (Sangon, Shanghai, China) and an automated blood cycler (Eppendorf, Hamburg, Germany). The procedure was carried out in strict accordance with the manufacturer’s recommended PCR conditions. The products were digested at 37 °C for 60 min and then digested with restriction endonuclease Eci I (New England Biolabs, Hitchin, UK) at 65 °C for 10 min to identify and cleave the variant sequences. The digested PCR products were resolved on a 2% agarose gel, stained with ethidium bromide, and then visualized under UV light (Figure 1).

### 2.3. Statistical Analysis

The appropriate two independent sample t-tests and Wilcoxon rank-sum tests were conducted to estimate differences in the general characteristics; plasma niacin levels; plasma niacinamide levels; and levels of enzymatic activity of NAMPT, NAPRT, and PARP-1 between healthy controls and EPL cases. For further analysis, the NAD-related continuous variables were divided into quartiles (Q1, Q2, Q3, Q4) according to the range of measured values. Univariate logistic regression was performed on the 18 variables related to the general characteristics and the diet of the subjects in order to avoid the influence of the correlations between the variables on the subsequent results and thus increase the reliability. *p* values < 0.10 (two-tailed) were considered to have statistical significance. Conditional logistic regression was used to assess the association between NAD-related enzymes and EPL risk, adjusting for eating speed, liquor drinking, and fresh fruit. The median of each group divided by quartiles for each variable was regarded as a continuous variable, and a linear trend test was performed. A *p* value < 0.05 (two-tailed) is a sign of statistical significance. The chi-square test was used to evaluate the association between PARP-1V762A gene polymorphism and EPL risk. GraphPad Prism version 8.0 was used as a graphing tool, and SPSS version 24.0 was used for statistical analysis.

## 3. Results

### 3.1. General Characteristics of the Subjects

There were 100 EPL cases (48% female, 64.45 ± 5.34 years old) and 100 matched healthy controls (48% female, 64.38 ± 5.09 years old) in the present study. No statistical difference was found in terms of body mass index (BMI) (cases: 23.72 ± 3.21 kg/m^2^, controls: 23.75 ± 3.32 kg/m^2^) and waist–hip ratio (WHR) (cases: 0.90 ± 0.05, controls: 0.89 ± 0.05) between the two groups. The basic information about smoking and drinking of the subjects has been previously neatened [28].

### 3.2. Relevant Variables for NAD of the Subjects

The plasma niacin level; the plasma nicotinamide level; and the concentrations of related protease NAMPT, NAPRT, and PARP-1 in healthy controls and EPL cases are shown in Table 1. The concentrations of NAPRT (*p* < 0.001) and PARP-1 (*p* = 0.001) in EPL cases were significantly lower than those in healthy controls. However, no significant difference was found in the level of plasma niacin, nicotinamide, or the concentration of NAMPT (*p* = 0.951, 0.732, and 0.767, respectively).

### 3.3. Association between Relevant Variables for NAD and Risk of EPL

**Figure 2:**
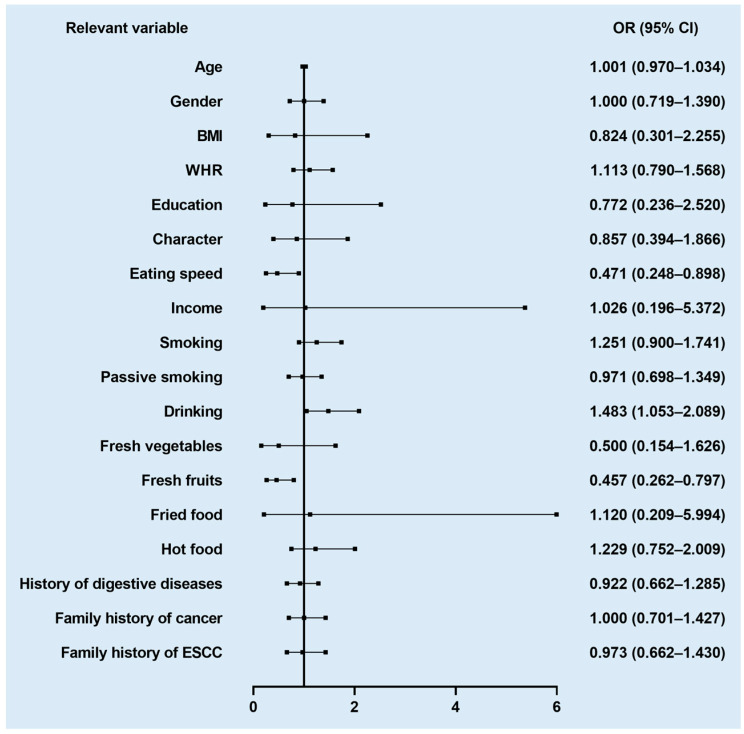
Forest plot of association between relevant variables and EPL risk.

Within this study, univariate logistic regression on 18 related variables was performed. According to their data characteristics, we adjusted the variables as either continuous (age), binary (gender, smoking, passive smoking, liquor drinking, a history of digestive disease, and a family history of cancer or ESCC), or multi-categorical (BMI, WHR, character, education, income, eating speed, fresh vegetables, fresh fruits, fried food, and hot food) variables. As shown in Figure 2, a total of three related variables were associated with the EPL risk, including drinking (*p* = 0.059), eating speed (*p* = 0.055), and fresh fruits (*p* = 0.021). Liquor drinking and faster eating speed were found to increase the EPL risk, while the intake of fresh fruit was negatively associated with the EPL risk. Several fruits that were consumed more frequently by the subjects were analyzed in our study, including citrus, orange, strawberry, pineapple, banana, and hawthorn.

As shown in Table 2, the levels of plasma niacin (*p* for trend = 0.632) and nicotinamide (*p* for trend = 0.804), and the concentration of NAMPT (*p* for trend = 0.641) showed no significant association with EPL risk after model adjustment. However, compared with the first quartile, the other three quartiles of NAPRT were positively associated with EPL risk (*p* = 0.014, 0.001, and 0.016, respectively). In addition, we found a positive linear relationship between the concentration of PARP-1 and EPL risk (*p* for trend = 0.021). Further analysis after gender differentiation revealed that this association could only be found in male subjects (Table 3).

### 3.4. The Gene Polymorphism of PARP-1 V762A

**Figure 3:**
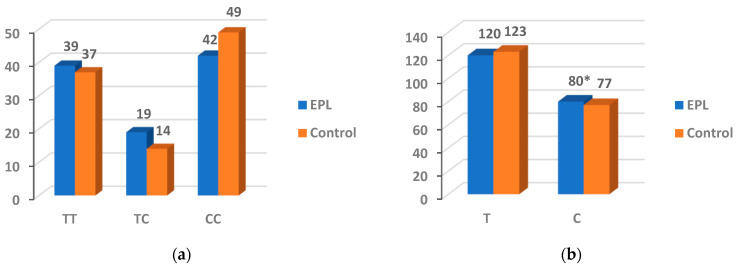
Genotype (**a**) and allele (**b**) frequencies for the PARP-1 V762A. * The difference was considered statistically significant (*p* < 0.05).

The proportions of TT, TC, and CC genotypes were 39%, 19%, and 42% in the EPL group, and 37%, 14%, and 49% in the control group (Figure 3). No statistical significance was observed with the genotypes between the two groups (*p* = 0.506 and 0.548, respectively). However, compared with control group, the frequency of the C allele was higher in the EPL group (*p* < 0.001).

## 4. Discussion

Cancer cells require a larger store of NAD than normal cells. Our study found that NAD is also used more frequently in EPL, as reflected by the over-expression of NAPRT and PARP-1, and the differential distribution frequency of the C allele in PARP-1 V762A.

Levels of plasma niacin and nicotinamide were not found to be associated with EPL risk. This may be related to the extensive and complex sources of niacin and nicotinamide involved in NAD synthesis in vivo. In addition to being ingested in the diet, niacin can also be converted from nicotinamide in the body. Shats et al. showed that intestinal flora can convert nicotinamide to niacin via microbial nicotinamidase (PncA), thereby utilizing the PH pathway to generate NAD [29]. Niacinamide can also be derived from food intake. In addition, it can be produced in certain reactions wherein NAD is used as a substrate for consumption [5,30,31]. This is one of the reasons why we did not perform a comparison of dietary niacin and niacinamide intakes in the subjects. Notably, previous studies have also failed to reveal the relationship between dietary niacin intake and esophageal cancer. Results of a case report showed improvement in esophageal histopathology after niacin supplementation in approximately half of the pellagra patients with acute esophagitis [32]. In addition, an Italian case–control study demonstrated an independent effect of niacin: niacin consumption was negatively related to the risk of esophageal cancer [33]. However, there is no shortage of studies negating the correlation between niacin and esophageal cancer [34,35]. This controversial result may be due to the differential expression of key enzymes for NAD synthesis, which allows niacin or nicotinamide to be absorbed and utilized inconsistently with expectations after entering the body from dietary intake.

The over-expression of NAPRT showed a significant positive correlation with the risk of EPL, which is consistent with our speculation based on studies related to esophageal cancer [36]. However, NAMPT was not detected to have the same correlation. This may be due to the ability of niacin to produce NAD more efficiently. In animal tissues, niacin is considered to be a better precursor of NAD than nicotinamide [37]. A study found that the addition of niacin, but not nicotinamide, increased NAD levels in cells by nearly double, and this was strongly correlated with the over-expression of NAPRT [38]. More importantly, the significant over-expression of NAPRT in EPL patients may be related to the major pathway of NAD synthesis in esophageal cancer tissues. A recent study by Chowdhry et al. found that esophageal cancer cells belong to the PH-amplified cell category and are completely dependent on the PH pathway in maintaining their NAD requirement [12]. This also confirms the rationale that the key enzyme of the salvage pathway, NAMPT, was not found to be over-expressed in EPL. Furthermore, the possibility that NAD in vivo is synthesized, bypassing the action of NAMPT in the presence of intestinal flora and PncA, cannot be ignored [29]. The non-linear relationship between the concentration of NAPRT and the EPL risk may be attributed to the interference of tryptophan [39]. Although many body tissues do not express the complete de novo pathway enzymes, NAD in the liver is primarily generated from tryptophan and is consumed to produce NAM, which is involved in the salvage pathway [31]. The role of tryptophan in the non-single pathways increases the complexity of NAD synthesis, which is likely to be ignored.

Table 3 found that the significant positive association between the concentration of NAPRT and EPL risk was only reflected in the male subgroup. This may be because men contribute more to smoking and drinking. A survey in the United States showed that adult men have a greater demand than women for all tobacco products, including cigarettes, hookahs, cigars, and e-cigarettes [40]. In addition, several studies have found that men drink more heavily and more frequently [41,42,43,44]. Preliminary studies from our laboratory in Huai’an District found that excessive smoking, passive smoking and liquor consumption all contribute to the increased risk of EPL [27]. Of course, the effect of hormones on the expression of NAPRT cannot be ruled out. On the one hand, androgen receptors were found to be more present in ESCC tissues and were associated with tumor differentiation, invasion, and lymph node metastasis [45]. An animal study showed that the highest incidence of ESCC is in male rats, followed by androgen-injected females [46]. On the other hand, Matsuoka et al. found that the proliferation of ESCC cell lines is accelerated by testosterone but inhibited by estradiol [47]. The anti-inflammatory capacity of estrogen and its regulation of lipid metabolism gives it the potential to delay the onset of esophageal cancer [48].

The over-expression of NAPRT in the case group demonstrates that EPL mainly ensures a large NAD requirement through the PH pathway. The linear positive correlation between PARP-1 and the EPL risk further fully validates that the surge of NAD is being fully utilized. Therefore, NAPRT inhibitors may be more functional, targeting the clinical treatment of EPL than NAMPT inhibitors. 2-Hydroxynicotinic acid (2-HNa), the first identified NAPRT inhibitor, can effectively silence NAPRT [49]. In recent years, the application of computer technology has identified chemical molecular formulas that are functionally similar to 2-HNa, promising the discovery of new NAPRT inhibitors for esophageal cancer therapy [50,51]. 

It remains difficult to show that PARP-1 variant genotypes increase susceptibility to EPL because no significant differences were found in the distribution of PARP-1 genotypes TT, TC, and CC between the two groups of subjects. However, the study by Hao et al. found that the PARP-1 variant C allele was associated with an increased risk of ESCC [52]. Our results of the variant C allele also reveal the possibility of its association with EPL risk, highlighting the possible impact of PARP-1 on ESCC susceptibility. Further studies need to be conducted to refine our findings.

## 5. Conclusions

In conclusion, we found that it is the over-expression of NAPRT, but not NAMPT, that provides the additional support for NAD synthesis in EPL and further ensures the oversupply of PARP-1. Our study is meaningful because it suggests that EPL and esophageal cancer are consistent in that both may meet the additional demand for NAD synthesis through the PH pathway. Hence, it may be possible to control the disease at the EPL stage. We believe that it is necessary to focus on subsequent NAPRT inhibitors research to promote the prevention and control of ESCC.

## Figures and Tables

**Table 1:** Comparison of NAD-related variables between two groups.

Median (25th–75th)	EPL	Controls	*p* Value
Niacin (nmol/L)	34.209 (30.238, 38.756)	32.488 (29.245, 41.422)	0.951
Nicotinamide (ng/mL)	0.613 (0.351, 1.042)	0.586 (0.371, 1.040)	0.732
NAPRT (pg/mL)	194.117 (152.068, 237.732)	146.972 (115.463, 219.051)	<0.001 *
NAMPT (pg/mL)	164.658 (142.565, 188.002)	160.528 (137.922, 190.628)	0.767
PARP-1 (pg/mL)	879.598 (669.161, 1294.424)	709.649 (431.848, 979.108)	0.001 *

* The difference was considered statistically significant (*p* < 0.05).

**Table 2:** ORs (95% CIs) of relevant variables for NAD with EPL.

	Q1	Q2	Q3	Q4	*p* for Trend
	OR (95%CI)	OR (95%CI)	OR (95%CI)	OR (95%CI)	
Niacin (nmol/L)	21.335–29.522	29.522–33.858	33.858–40.162	40.162–395.435	
EPL cases (%)	46.94	42.86	69.39	40.82	
crude model	1	0.913 (0.505–1.650)	1.478 (0.871–2.509)	0.870 (0.478–1.583)	0.642
adjusted model	1	0.970 (0.534–1.763)	1.341 (0.780–2.304)	0.886 (0.484–1.620)	0.632
Nicotinamide (ng/mL)	0.070–0.362	0.362–0.591	0.591–1.039	1.039–37.700	
EPL cases (%)	52.00	46.00	52.00	50.00	
crude model	1	0.885 (0.505–1.550)	1.000 (0.581–1.722)	0.962 (0.555–1.665)	0.999
adjusted model	1	1.063 (0.597–1.894)	1.071 (0.615–1.866)	1.090 (0.615–1.931)	0.804
NAMPT (pg/mL)	65.119–141.113	141.113–161.586	161.586–188.603	188.603–776.568	
EPL cases (%)	42.86	52.08	57.14	47.92	
crude model	1	1.215 (0.680–2.171)	1.333 (0.757–2.348)	1.118 (0.619–2.020)	0.819
adjusted model	1	1.221 (0.680–2.190)	1.347 (0.760–2.387)	1.192 (0.655–2.170)	0.641
NAPRT (pg/mL)	44.12–131.20	131.21–171.67	171.68–233.12	233.132065.36	
EPL cases (%)	20.41	54.16	73.47	52.08	
crude model	1	2.654 (1.280–5.504) *	3.600 (1.787–7.254) *	2.552 (1.226–5.314) *	0.117
adjusted model	1	2.526 (1.208–5.282) *	3.268 (1.597–6.685) *	2.477 (1.187–5.170) *	0.132
PARP-1 (pg/mL)	133.53–537.78	537.79–790.48	790.49–1070.71	1070.71–3099.21	
EPL cases (%)	37.50	40.43	56.25	65.96	
crude model	1	1.078 (0.566–2.054)	1.500 (0.826–2.723)	1.759 (0.984–3.144)	0.033
adjusted model	1	0.987 (0.510–1.908)	1.376 (0.753–2.517)	1.816 (1.011–3.262) *	0.021 *

* The difference was considered statistically significant (*p* < 0.05).

**Table 3:** ORs (95% CIs) of relevant variables for NAD with EPL in male and female subgroups.

	Q1	Q2	Q3	Q4	*p* for Trend
	OR (95%CI)	OR (95%CI)	OR (95%CI)	OR (95%CI)	
Male					
Niacin (nmol/L)					
EPL cases (%)	54.55	38.46	72.00	37.93	
Adjusted OR (95%CI)	1	0.728 (0.306–2.610)	1.229 (0.579–2.610)	0.703 (0.301–1.645)	0.483
Nicotinamide (ng/mL)					
EPL cases (%)	50.00	44.44	44.44	58.62	
Adjusted OR (95%CI)	1	0.927 (0.387–2.220)	0.856 (0.391–1.877)	1.251 (0.614–2.549)	0.454
NAMPT (pg/mL)					
EPL cases (%)	48.00	50.00	53.85	44.44	
Adjusted OR (95%CI)	1	1.165 (0.513–2.648)	1.106 (0.499–2.451)	1.019 (0.444–2.339)	0.949
NAPRT (pg/mL)					
EPL cases (%)	21.74	48.15	81.48	43.48	
Adjusted OR (95%CI)	1	2.373 (0.820–0.869)	3.464 (1.287–9.319) *	2.143 (0.723–6.350)	0.401
PARP-1 (pg/mL)					
EPL cases (%)	31.25	37.04	76.92	67.65	
Adjusted OR (95%CI)	1	1.355 (0.452–4.056)	1.709 (0.600–4.866)	2.619 (0.972–7.062)	0.020 *
Female					
Niacin (nmol/L)					
EPL cases (%)	40.74	47.83	66.67	45.00	
Adjusted OR (95%CI)	1	1.237 (0.515–2.973)	1.411 (0.614–3.240)	1.196 (0.473–3.024)	0.815
Nicotinamide (ng/mL)					
EPL cases (%)	55.00	46.88	60.87	38.10	
Adjusted OR (95%CI)	1	1.304 (0.496–3.432)	1.503 (0.564–4.010)	1.025 (0.357–2.942)	0.783
NAMPT (pg/mL)					
EPL cases (%)	37.50	50.00	60.87	52.38	
Adjusted OR (95%CI)	1	1.233 (0.525–2.896)	1.507 (0.630–3.607)	1.312 (0.528–3.260)	0.575
NAPRT (pg/mL)					
EPL cases (%)	19.23	61.90	63.64	60.00	
Adjusted OR (95%CI)	1	2.373 (0.820–6.869)	3.464 (1.287–9.319) *	2.143 (0.723–6.350)	0.261
PARP-1 (pg/mL)					
EPL cases (%)	40.63	45.00	60.87	61.54	
Adjusted OR (95%CI)	1	0.880 (0.352–2.203)	1.254 (0.572–2.750)	1.576 (0.646–3.848)	0.278

* The difference was considered statistically significant (*p* < 0.05).

## Data Availability

All data are available with the consent of the corresponding author.
